# Synthesis and cellular effects of novel 1,3,5-triazine derivatives in DLD and Ht-29 human colon cancer cell lines

**DOI:** 10.1007/s10637-019-00838-9

**Published:** 2019-09-13

**Authors:** Agnieszka Wróbel, Beata Kolesińska, Justyna Frączyk, Zbigniew J. Kamiński, Anna Tankiewicz-Kwedlo, Justyna Hermanowicz, Robert Czarnomysy, Dawid Maliszewski, Danuta Drozdowska

**Affiliations:** 1 Department of Organic Chemistry, Medical University of Bialystok, Białystok, Poland; 2 Institute of Organic Chemistry, Lodz University of Technology, Lodz, Poland; 3 Department of Monitored Pharmacotherapy, Medical University of Bialystok, Białystok, Poland; 4 Department of Clinical Pharmacy, Medical University of Bialystok, Bialystok, Poland; 5 Department of Synthesis and Technology of Drugs, Medical University of Bialystok, Bialystok, Poland

**Keywords:** Colon cancer, Hybrid anticancer drugs, Triazine derivatives, Nitrogen mustards, Apoptosis, Drug design

## Abstract

This study provides new information on the cellular effects of 1,3,5-triazine nitrogen mustards with different peptide groups in DLD and Ht-29 human colon cancer cell lines. A novel series of 2,4,6-trisubstituted 1,3,5-triazine derivatives bearing 2-chloroethyl and oligopeptide moieties was designed and synthesized**.** The most cytotoxic derivative was triazine with an Ala-Ala-OMe substituent on the ring (compound **7b**). This compound induced time- and dose-dependent cytotoxicity in the DLD-1 and HT-29 colon cancer cell lines. The triazine derivative furthermore induced apoptosis through intracellular signaling pathway attenuation. Compound **7b** may be a candidate for further evaluation as a chemotherapeutic agent against colorectal cancer.

## Introduction

Nitrogen mustards (NMs), such as chlorambucil and mechlorethamine, are an extensively investigated and clinically used class of anticancer drugs. Although they are themselves highly carcinogenic, these bifunctional alkylating agents can be used in the treatment of various cancers and autoimmune diseases [1–4]***.*** Their mechanism of activity is connected to their ability to cross-link double strands of DNA involving the distal guanine bases in the opposite strand of 5’-GNC sequences, yielding bifunctional DNA lesions. This eventually inhibits tumor growth and the proliferation of cancer cells, by impeding the replication and transcription process [5–8]. New analogues of nitrogen mustards with increased selectivity and pharmacological effectiveness are continually sought [9].

Bifunctional hybrids of triazine nitrogen mustards are a unique group of compounds with confirmed anti-proliferative activity. Recent research suggests that derivatives with one, two or three fragments bearing the 2-chloroethylamine moiety characteristic for nitrogen mustards introduced into the 1,3,5-triazine scaffold show anticancer ability [10, 11]. Compounds containing a melamine fragment in the structure inhibit a wide range of enzymes involved in the transcription and translation process. The main common feature of these compounds is the presence of a triazine ring substituted by alkyl-(aryl)-amino residues. A number of studies have identified many new, highly potent agents which include a 1,3,5-triazine core. It has been reported that these compounds show inhibitory activity against important enzymes involved in cell proliferation cycles.

Structure-activity studies have revealed that one of the molecular targets is phosphatidylinositol 3-kinases (PI3K). The melamine derivative ZSTK474 [2-(2difluoromethylbenzimidazol-1-yl)-4,6-dimorpholino-1,3,5-triazine] demonstrates strong antitumor activity against human cancer and reduces the growth of tumor cells by inhibiting PI3K [12–14]. It has been also reported that the use of 2,4-diamino-6-(pyridine-4-yl)-1,3,5-triazine (4PyDAT) impedes the process of angiogenesis [15]. Furthermore, molecules based on a structurally modified triazine ring have been found to inhibit histone deacetylase and topoisomerases, as well as blocking telomere replication and stabilizing telomeric G-rich single-stranded DNA (G-quadruplex), causing G2/M arrest and apoptosis with the possible involvement of p53 [16–20]. Recently, many compounds containing a 1,3,5-triazine ring have been obtained which have potential pharmacological effects. As only two of these derivatives have been tested clinically so far, there is rich potential for finding new therapeutic compounds in this group [21].

In our previous paper, we presented the synthesis of triazine derivatives bearing one, two or three 2-chloroethylamine residues. The cytotoxicity of all the obtained compounds, as well as the apoptotic index and cell viability, were studied for the standard cell line of mammalian tumor MCF-7. All of the obtained triazines inhibited colony formation by 50% at concentrations in the range of 13.88 to 146.79 μM. As a comparison, the IC_50_ of chlorambucil was 24.6 μM. In addition, it was found that the triazine analogues had anti-proliferative activity against prostate cancer LNCaP, lung cancer A549, breast cancer T47D, colorectal cancer SW707 and Jurkat lymphoblastic leukemia. A significant finding was that in the case of estrogen-dependent breast cancer MCF-7, which is resistant to chemotherapy, activity increased with the number of 2-chloroethyloamino moieties [22]. It was found that the compounds with antitumor cytotoxicity towards the standard cell line of mammalian tumor MCF-7 were strongly alkylating agents, which reacted easily with most of the nucleophilic functional groups typical for proteins and nucleic acids [23]**.**

**Fig. 1:**
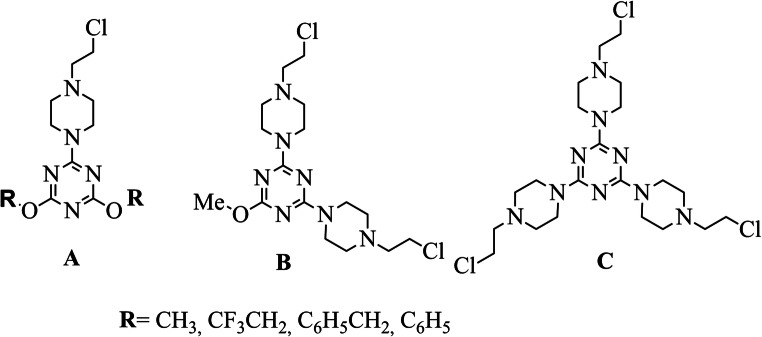
Structures of triazine mustards

In our further studies, dual hybrid molecules were synthesized by the functionalization of a melamine scaffold with 2-chloroethyloamino (one, two or three) fragments attached to 1,3,5- triazine via the piperazine ring (Fig. 1). The anti-proliferative activity of the compounds was evaluated using two tumor cell lines: MCF-7 and MDA-MB-231. The results confirmed the inhibitory effect of the tested compounds on MCF-7 cancer cells. This effect was dependent on the structure of the substituents on the 1,3,5- triazine ring [24]**.** It was also found that 1,3,5-triazine with three alkylating substituents induced time- and dose-dependent cytotoxicity in LBC3, LN-18 and LN-229 glioma cell lines. Compound **C** caused apoptosis, necrosis and strong cell shrinkage, reducing the number of apoptotic bodies. It can therefore be considered as a candidate for further evaluation as a chemotherapeutic agent against glioma cells [25].

## Materials and methods

### Chemistry

#### General information

Thin layer chromatography experiments (TLC) were carried out on silica gel (Merck; 60 Å F254). Spots were located with UV light (254 and 366 nm) and 1% ethanolic 4-(4-nitrobenzyl) pyridine (NBP). Analytical RP-HPLC was performed on a Waters 600S HPLC system (Waters 2489 UV/VIS detector, Waters 616 pump, Waters 717 plus autosampler, HPLC manager software from Chromax) using a Vydac C18 column (25 cm × 4.6 mm, 5 mm; Sigma). HPLC was performed with a gradient of 0.1% TFA in H_2_O (A) and 0.08% TFA in MeCN (B), at a flow rate of 1 mL/min with UV detection at 220 nm, t_R_ in min.

MS analysis was performed on an MS Bruker micrOTOF-QIII.

Infrared spectra were recorded as KBr pellets or on film using a Bruker ALPHA spectrometer or a PerkinElmer Spectrum 100.

^1^H-NMR and ^13^C-NMR, spectra were recorded on a Bruker Avance DPX 250 (250 MHz) spectrometer and Varian (300 MHz). Chemical shifts (ppm) were relative to TMS, used as an internal standard. Multiplicities are marked as s = singlet, d = doublet, t = triplet, q = quartet, qu = quintet, m = multiplet.

#### Preparation of dipeptides

##### Boc-Aib-Ala-OMe (1); typical procedure

Boc-Aib-OH (0.406 g, 2 mmol) and NMM (0.110 mL, 1 mmol) were added to a vigorously stirred solution of DMT/NMM/TosO^−^ (0.826 g, 2 mmol) in CH_2_Cl_2_ (10 mL) cooled to 0 °C. Stirring was continued until the condensing reagent disappeared (TLC analysis, staining with 0.5% solution of NBP) (1 h), after which HCl*Ala-OMe (0.278 g, 2 mmol) and NMM (0.220 mL, 2 mmol) were added. The mixture was stirred for an additional 2 h at 0 °C and left overnight at room temperature. The mixture was diluted with CH_2_Cl_2_ (10 mL), then the solution was washed successively with water, 0.5 M aqueous NaHSO_4_, water, 0.5 M aqueous NaHCO_3_ and water again. The organic layer was dried with Na_2_SO_4_, filtered and concentrated to dryness. The residue was dried under a vacuum with P_2_O_5_ and KOH to constant weight, to obtain the neutral peptide. Finally, 0.559 g was obtained, corresponding to a 97% yield.

IR (film): ν = 3678, 3608, 3430, 3017, 2965, 2398, 1734, 1672, 1512, 1451, 1420 [cm^−1^].

^1^H-NMR (250 MHz; CDCl_3_) δ = 1.39 (s,9H, (CH_3_)_3_-C-); 1.41 (d, 3H, J = 7.2 Hz, CH_3_ CH-); 1.57 (s, 6H, (CH_3_)_2_-C-); 3.72 (s, 3H, CH_3_O-); 4.36 (q, 1H, J = 7.2 Hz, CH_3_-CH-) [ppm].

Anal. RP-HPLC (10–97%B in 30 min): *t*_R_ = 9.55 min (purity 98.8%).

##### Boc-Ala-Ala-OMe (1b)

Starting materials: Boc-Ala-OH (0.378 g, 2 mmol), HCl*Ala-OMe (0.278 g, 2 mmol), DMT/NMM/TosO^−^ (0.826 g, 2 mmol), NMM (0.330 mL, 3 mmol). Product: Boc-Ala-Ala-OMe. Activation time: 1 h, yield 0.527 g (96%).

IR (film): ν = 3119, 3008, 1740, 1718, 1675, 1504, 1450, 1376, 1314 [cm^−1^].

^1^H-NMR (250 MHz; CDCl_3_) δ = 1.36 (d, 3H, J = 6.8 Hz, CH_3_-CH-); 1.41 (d, 3H, J = 6.8 Hz, CH_3_ CH-); 1.45 (s, 9H, (CH_3_)_3_-C-); 3.75 (s, 3H, CH_3_O-); 4.57 (q, 1H, J = 6.8 Hz, CH_3_ CH-); 4.61 (q, 1H, J = 6.8 Hz, CH_3_ CH-); 4.97 (1H, bs, -NH-), 6.49 (1H, bs, -NH-) [ppm].

Anal. RP-HPLC (10–97%B in 30 min): *t*_R_ = 9.03 min (purity 98.1%).

##### Boc-Ser-Ala-OMe (1c)

Starting materials: Boc-Ser-OH (0.410 g, 2 mmol), HCl*Ala-OMe (0.278 g, 2 mmol), DMT/NMM/TosO^−^ (0.826 g, 2 mmol), NMM (0.330 mL, 3 mmol). Product: Boc-Ser-Ala-OMe. Activation time: 1.5 h, yield 0.540 g (93%).

IR (film): ν = 3110, 3002, 1748, 1722, 1648, 1501, 1444, 1372, 1314 [cm^−1^].

^1^H-NMR (250 MHz; CDCl_3_) δ =1.38 (d, 3H, J = 7.12 Hz, CH_3_-CH-); 1.43 (s, 9H, (CH_3_)_3_-C-); 3.65 (dd, 2H, J_1_ = 6.88 Hz, J_2_ = 3.22 Hz, HO-CH_2_-); 3.72 (s, 3H, CH_3_O-); 4.35 (q, 1H, J = 7.1 Hz, CH_3_-CH-); 4.37 (t, 1H, J = 7.2 Hz, -CH-CH2-); 4.55 (t, 1H, J = 6.88 Hz, -CH_2_-CH-) [ppm].

Anal. RP-HPLC (10–97%B in 30 min): *t*_R_ = 7.25 min (purity 98.7%).

##### Fmoc-Lys(Boc)-Ala-OMe (1d)

Starting materials: Fmoc-Lys(Boc)-OH (0.937 g, 2 mmol), HCl*Ala-OMe (0.278 g, 2 mmol), DMT/NMM/TosO^−^ (0.826 g, 2 mmol), NMM (0.330 mL, 3 mmol). Product: Fmoc-Lys(Boc)-Ala-OMe. Activation time: 1 h, yield 1.041 g (94%).

IR (film): ν = 3401, 3087, 2994, 1753, 1748, 1710, 1674, 1546, 1452, 1373 [cm^−1^].

^1^H-NMR (250 MHz; CDCl_3_) δ = 1.41 (d, 3H, J = 7.2 Hz, CH_3_-CH-); 1.43 (s, 9H, (CH_3_)_3_C-); 1.48–1.55 (m, 4H, -CH_2_-CH_2_-); 1.88 (qu, 2H, J = 7.4 Hz, -CH-CH_2_-); 3.21 (t, 2H, J = 7.0 Hz, NH-CH_2_-); 3.71 (s, 3H, CH_3_O-); 4.36 (q, 1H, J = 7.2 Hz, CH_3_-CH-); 4.46 (t, 1H, J = 3.1 Hz, -CH-CH_2_-); 4.62 (d, 2H, J = 3.1 Hz, -CH_2_-O-); 7.36–8.11 (m, 8H, arom.) [ppm].

Anal. RP-HPLC (10–97%B in 30 min): *t*_R_ = 10.25 min (purity 98.1%).

##### Fmoc-Asp(OtBu)-Ala-OMe (1e)

Starting materials: Fmoc-Asp(OtBu)-OH (0.823 g, 2 mmol), HCl*Ala-OMe (0.278 g, 2 mmol), DMT/NMM/TosO^−^ (0.826 g, 2 mmol), NMM (0.330 mL, 3 mmol). Product: Fmoc-Asp(OtBu)-Ala-OMe. Activation time: 1 h, yield 0.934 g (94%).

IR (film): ν = 3410, 3090, 2990, 1750, 1740, 1710, 1675, 1540, 1450, 1375 [cm^−1^].

^1^H-NMR (250 MHz; CDCl_3_) δ = 1.40 (d, 3H, J = 7.1 Hz, CH_3_- CH-); 1.46 (s, 9H, -OC(CH_3_)_3_); 2.57 (dd, 1H, J_1_ = 17.2 Hz, J_2_ = 7.3 Hz, -OCO-CH_2_-CH-); 2.92 (dd, 1H, J_1_ = 17.2 Hz, J_2_ = 4.1 Hz, -OCO-CH_2_-CH-); 3.73 (s, 3H, CH_3_O-); 4.26 (t, 1H, J = 7.1 Hz, -CH-CH_2_O-); 4.42 (d, 2H, J = 7.1 Hz, -CH-CH_2_O-); 4.48–4.60 (m, 2H, CH_3_CH- + OCO-CH_2_-CH-); 5.97 (d, 1H, J = 7 Hz, NH); 7.07 (d, 1H, J = 5 Hz, NH); 7.31 (t, 2H, J = 7 Hz, arom.); 7.41 (t, 2H, J = 7 Hz, arom.); 7.58 (d, 2H, J = 7 Hz, arom.); 7.77 (d, 2H, J = 7 Hz, arom.) [ppm].

Anal. RP-HPLC (10–97%B in 30 min): *t*_R_ = 10.05 min (purity 97.9%).

##### Fmoc-Trp(Boc)-Ala-OMe

Starting materials: Fmoc-Trp(Boc)-OH (1.053 g, 2 mmol), HCl*Ala-OMe (0.278 g, 2 mmol), DMT/NMM/TosO^−^ (0.826 g, 2 mmol), NMM (0.330 mL, 3 mmol). Product: Fmoc-Trp(Boc)-Ala-OMe. Activation time: 1 h, yield 1.187 g (97%).

IR (film): ν = 3412, 3088, 2987, 1754, 1743, 1711, 1679, 1538, 1452, 1375 [cm^−1^].

^1^HNMR (250 MHz; CDCl_3_) δ = 1.41 (d, 3H, J = 7.2 Hz, CH_3_-CH-); 1.43 (s, 9H, (CH_3_)_3_C-); 3.15 (d, 2H, J = 6.7 Hz, -CH-CH_2_-); 3.71 (s, 3H, CH_3_O-); 4.91 (t, 1H, J = 6.7 Hz, CH-CH_2_-); 4.36 (q, 1H, J = 7.2 Hz, CH_3_-CH-); 4.46 (t, 1H, J = 3.1 Hz, -CH-CH_2_-); 4.62 (d, 2H, J = 3.1 Hz, -CH_2_-O-); 7.03–8.11 (m, 13H, arom.) [ppm].

Anal. RP-HPLC (10–97%B in 30 min): *t*_R_ = 11.14 min (purity 98.1%).

#### Removal of protected groups

##### Boc group deprotection

A Boc-protected dipeptide solution (1 mmol) dissolved in 1,4-dioxane (5 mL) was cooled in a water-ice bath with vigorously stirring, and a 4 M solution of HCl in 1,4-dioxane (5 mL) was added. Stirring was continued for 4 h. The solution was then removed in a vacuum evaporator and 1,4-dioxane (10 mL) was added to the residue. The solvent was removed again using a vacuum evaporator. This operation was repeated four times. The residue was dried in a vacuum desiccator to constant weight. The procedure gave HCl*Aib-Ala-OMe, HCl*Ala-Ala-OMe and HCl*Ser-Ala-OMe in quantitative yield. The derivatives obtained were used in the subsequent reaction steps without additional purification procedures.

##### Fmoc-group deprotection

A solution of Fmoc-protected dipeptide derivatives (1 mmol) dissolved in DCM (5 mL) was cooled in a water-ice bath with intensive stirring and a 25% piperidine solution in DCM (10 mL) was added. Stirring was continued for 15 min, then MeOH (20 ml) was added to the solution. Stirring was continued for 5 min. The solvent was removed using a vacuum evaporator. To the residue was added 10 mL of DCM and the solvent was removed again in a vacuum evaporator. This operation was repeated four times. The solid residue was dried under a vacuum to constant weight. The procedure gave H-Lys(Boc)-Ala-OMe, H-Asp(OtBu)-Ala-OMe and H-Trp(Boc)-Ala-OMe in quantitative yield. The derivatives obtained were used immediately in subsequent reaction steps, without additional purification procedures.

#### Synthesis of **4a-f** from DCMT and dipeptides

##### Synthesis of 2-chloro-4-methoxy-6-(NH-Aib-Ala-OMe)-1,3,5-triazine (4a). General procedure

To a vigorously stirred slurry of solid NaHCO_3_ (0.42 g, 5 mmol) in dichloromethane (DCM) (3 mL) cooled to 0 °C was added 2,4-dichloro-6-methoxy-1,3,5-triazine (DCMT) (0.270 g, 1.5 mmol) in DCM (3 mL), followed by the slow addition of a solution of HCl*NH-Aib-Ala-OMe (0.337 g, 1.5 mmol). Stirring was continued until complete consumption of the DCMT, as shown by the disappearance of a DCMT spot stained with 0.5% solution of NBP in ethanol at room temperature, monitored by TLC analysis. After filtration of the solid deposit, the solvent was removed using vacuum evaporation and the residue dried under a vacuum with P_2_O_5_ and KOH to a constant weight, giving 0.491 g of 2-chloro-4-methoxy-6-(NH-Aib-Ala-OMe)-1,3,5-triazine (**4a**), yield = 98.7% as colorless oil.

Analysis: for C_12_H_18_ClN_5_O_4_ (331.76): calculated: C 43.4%, H 5.5%, Cl 10.7%, N 21.1%, found: C 43.2%, H 5.6%, Cl 10.7%, N 21.2%.

^1^H-NMR (250 MHz; CDCl_3_) δ = 1.36 (s, 6H, ((CH_3_)_2_-C-); 1.41 (s, 3H, d, J = 7.2 Hz, CH_3_-CH-); 3.72 (s, 3H, CH_3_O-); 4.02 (s, 3H, CH_3_O-); 4.36 (q, 1H, J = 7.2 Hz, CH_3_-CH-) [ppm].

^13^C-NMR (75 MHz, CDCl_3_): δ = 17.7, 27.1, 48.1, 52.3, 54.6, 58.1, 159.7, 166.0, 172.9, 175.6 [ppm].

##### Synthesis of 2-chloro-4-methoxy-6-(NH-Ala-Ala-OMe)-1,3,5-triazine (4b)

Starting material: solid NaHCO_3_ (0.42 g, 5 mmol), DCMT (0.270 g, 1.5 mmol), HCl*H-Ala-Ala-OMe (0.316 g, 1.5 mmol). Product: 0.465 g 2-chloro-4-methoxy-6-(NH-Ala-Ala-OMe)-1,3,5-triazine (**4b**), yield = 97.6%, colorless oil.

Analysis: for C_11_H_16_ClN_5_O_4_ (317.73): calculated: C 41.6%, H 5.1%, Cl 11.2%, N 22%, found: C 41.5%, H 5.2%, Cl 11.2%, N 22.1%.

^1^H-NMR (250 MHz; CDCl_3_) δ = 1.39 (d, 3H, J = 7.0 Hz, CH_3_-CH-); 1.41 (d, 3H, J = 7.2 Hz, CH_3_-CH-); 3.71 (s, 3H, CH_3_O-); 4.02 (s, 3H, CH_3_O-); 4.35 (q, 1H, J = 7.2 Hz, CH_3_-CH-); 4.38 (q, 1H, J = 7.0 Hz, CH_3_-CH-) [ppm].

^13^C-NMR (75 MHz, CDCl_3_): δ = 17.4, 17.6, 47.8, 49.2, 52.3, 54.6, 159.8, 166.0, 172.2, 172.8 [ppm].

##### Synthesis of 2-chloro-4-methoxy-6-(NH-Ser-Ala-OMe)-1,3,5-triazine (4c)

Starting material: solid NaHCO_3_ (0.42 g, 5 mmol), DCMT (0.270 g, 1.5 mmol), HCl*H-Ser-Ala-OMe (0.340 g, 1.5 mmol). Product: 0.480 g 2-chloro-4-methoxy-6-(NH-Ser-Ala-OMe)-1,3,5-triazine (**4c**), yield = 95.8%, colorless oil.

Analysis: for C_11_H_16_ClN_5_O_5_ (333.73): calculated: C 39.6%, H 4.8%, Cl 10.6%, N 21%, found: C 39.7%, H 4.7%, Cl 10.6%, N 21%.

^1^H-NMR (250 MHz; CDCl_3_) δ = 1.41 (d, 3H, J = 7.2 Hz, CH_3_-CH-); 3.67 (dd, 2H, J_1_ = 6.8 Hz, J_2_ = 3.2 Hz, -CH-CH_2_-OH); 3.78 (s, 3H, CH_3_O-); 4.05 (s, 3H, CH_3_O-); 4.35 (1, 1H, J = 7.2 Hz, CH_3_-CH-); 4.48 (t, 1H, J = 6.8 Hz, -CH-CH_2_-) [ppm].

^13^C-NMR (75 MHz, CDCl_3_): δ = 17.6, 38.3, 47.8, 52.3, 54.6, 61.4, 159.5, 166.0171.3, 172.9 [ppm].

##### Synthesis of 2-chloro-4-methoxy-6-(NH-Lys(Boc)-Ala-OMe)-1,3,5-triazine (4d)

Starting material: solid NaHCO_3_ (0.42 g, 5 mmol), DCMT (0.270 g, 1.5 mmol), H-Lys(Boc)-Ala-OMe (0.497 g, 1.5 mmol). Product: 0.685 g 2-chloro-4-methoxy-6-(NH-Lys(Boc)-Ala-OMe)-1,3,5-triazine (**4d**), yield = 96.2%, colorless oil.

Analysis: for C_19_H_31_ClN_6_O_6_ (474.94): calculated: C 48%, H 6.6%, Cl 7.5%, N 17.7%, found: C 48.1%, H 6.7%, Cl 7.4%, N 17.6%.

^1^H-NMR (250 MHz; CDCl_3_) δ = 1.26–1.57 (m, 4H, -CH_2_-CH_2_-); 1.41 (d, 3H, d, J = 7.2 Hz, CH_3_-CH-); 1.43 (s, 9H, (CH_3_)_3_C-); 1.93 (dt, 2H, J_1_ = 7.5 Hz, J_2_ = 5.1 Hz, -CH-CH_2_-); 3.22 (t, 2H, J = 7.1 Hz, -CH_2_-NH-); 3.71 (s, 3H, CH_3_O-); 4.05 (s, 3H, CH_3_O-); 4.28 (t, 1H, J = 7.5 Hz, -CH-CH_2_-); 4.36 (q, 1H, J = 7.2 Hz, CH_3_-CH-) [ppm].

^13^C-NMR (75 MHz, CDCl_3_): δ = 17.7, 21.4, 28.2, 29.3, 29.6, 40.5, 47.8, 52.3, 53.6, 54.6, 80.5, 156.3, 159.5, 166.0, 172.5, 172.9 [ppm].

##### Synthesis of 2-chloro-4-methoxy-6-(NH-Asp(OtBu)-Ala-OMe)-1,3,5-triazine (4e)

Starting material: solid NaHCO_3_ (0.42 g, 5 mmol), DCMT (0.270 g, 1.5 mmol), H-Asp(OtBu-Ala-OMe (0.411 g, 1.5 mmol). Product: 0.607 g 2-chloro-4-methoxy-6-(NH-Asp(OtBu)-Ala-OMe)-1,3,5-triazine (**4e**), yield = 96.9%, colorless oil.

Analysis: for C_16_H_24_ClN_5_O_6_ (417.84): calculated: C 46%, H 5.8%, Cl 8.5%, N 16.8%, found: C 46%, H 5.9%, Cl 8.5%, N 16.8%.

^1^H-NMR (250 MHz; CDCl_3_) δ = 1.38 (s, 9H, (CH_3_)_3_C-); 1.41 (d, 3H, J = 7.2 Hz, CH_3_-CH-); 3.01 (d, 2H, J = 6.3 Hz, -CH-CH_2_-); 3.72 (s, 3H, CH_3_O-); 4.02 (s, 3H, CH_3_O-); 4.36 (1, 1H, J = 7.2 Hz, CH_3_-CH-); 5.02 (t, 1H, J = 6.3 Hz, -CH-CH_2_-) [ppm].

^13^C-NMR (75 MHz, CDCl_3_): δ = 17.7, 28.1, 37.9, 47.8, 52.3, 53.6, 54.6, 81.4, 159.6, 166.0, 172.3, 172.5, 172.9 [ppm].

##### Synthesis of 2-chloro-4-methoxy-6-(NH-Trp(Boc)-Ala-OMe)-1,3,5-triazine (4f)

Starting material: solid NaHCO_3_ (0.42 g, 5 mmol), DCMT (0.270 g, 1.5 mmol), H-Trp(Boc)-Ala-OMe (0.584 g, 1.5 mmol). Product: 0.776 g 2-chloro-4-methoxy-6-(NH-Trp(Boc)-Ala-OMe)-1,3,5-triazine (**4f**), yield = 97.1%, colorless oil.

Analysis: for C_24_H_29_ClN_6_O_6_ (532.98): calculated: C 54.1%, H 5.5%, Cl 6.7%, N 15.8%, found: C 54%, H 5.4%, Cl 6.7%, N 15.7%.

^1^H-NMR (250 MHz; CDCl_3_) δ = 1.41 (d, 3H, J = 7.2 Hz, CH_3_-CH); 1.44 (s, 9H, (CH_3_)_3_C-); 3.15 (d, 2H, J = 6.7 Hz, -CH-CH_2_-); 3.71 (s, 3H, CH_3_O-); 4.02 (s, 3H, CH_3_O-); 4.37 (q, 1H, J = 7.2 Hz, CH_3_-CH-); 4.91 (t, 1H, J = 6.7 Hz, CH-CH_2_-); 7.03–7.98 (m, 5H, arom) [ppm].

^13^C-NMR (75 MHz, CDCl_3_): δ = 17.7, 27.7, 28.2, 47.9, 52.3, 53.6, 54.6, 80.6, 115.4, 117.1, 118.8, 120.1, 122.8, 123.7, 129.6, 135.4, 149.2, 166.0, 162.1, 172.3, 172.9 [ppm].

#### Synthesis of 2-[4-(2-chloroethyl)piperazin-1-yl]-4-methoxy-6-(NH-dipeptydyl)-1,3,5-triazines **7a**-**f**

##### Synthesis of 2-[4-(2-chloroethyl)piperazin-1-yl]-4-methoxy-6-(NH-Aib-Ala-OMe)-1,3,5-triazine (7a). General procedure

To a vigorously stirred solution of 2-chloro-4-methoxy-6-(NH-Aib-Ala-OMe)-1,3,5-triazine (**4a**) (0.332 g, 1 mmol) in DCM (10 mL) cooled to 0 °C in an ice-water bath was added 1,4-diazabicyclo[2.2.2]octane (DABCO, **5**) (0.112 g, 1 mmol). Stirring at 0 °C was continued until complete consumption of **4a**, as shown by TLC analysis (Rf = 0.45, DCM; staining with 0.5% solution of NBP in ethanol). The cooling bath was removed and stirring was continued at room temperature until all **6a** salt was consumed, as shown by TLC analysis (Rf = 0.00, pure DCM, colored with 0.5% solution of NBP in ethanol). The solvent was removed by evaporation and the residue was dried under a vacuum with P_2_O_5_ and KOH to constant weight, giving 0.442 g 2-[4-(2-chloroethyl) piperazin-1-yl]-4-methoxy-6-(NH-Aib-Ala-OMe)-1,3,5-triazine (**7a**), yield = 99.1%, colorless oil.

Analysis: for C_18_H_30_ClN_7_O_4_ (443.93): calculated: C 48.7%, H 6.8%, Cl 8%, N 22.1%, found: C 48.6%, H 6.7%, Cl 8%, N 22%.

^1^H-NMR (250 MHz; CDCl_3_) δ = 1.36 (s, 6H, (CH_3_)_2_-C-); 1.41 (d, 3H, J = 7.2 Hz, CH_3_-CH-); 2.66 (t, 2x2H, J = 7.8 Hz, -N-CH_2_-); 2.85 (t, 2H, J = 6.2 Hz, -N-CH_2_-); 3.60 (t, 2x2H, J = 7.8 Hz, -N-CH_2_-); 3.86 (2, 2H, J = 6.2 Hz, Cl-CH_2_-); 3.71 (s, 3H, CH_3_O-); 4.02 (s, 3H, CH_3_O-); 4.36 (q, 1H, J = 7.2 Hz, CH_3_-CH-) [ppm].

^13^C-NMR (75 MHz, CDCl_3_): δ = 17.7, 27.1, 41.2, 44.1, 48.1, 51.8, 52.3, 53.1, 54.6, 58.1, 167.8, 171.7, 172.9, 175.6 [ppm].

LC/MS: 444.9373 ([M + H]^+^, C_18_H_31_ClN_7_O_4_^+^; calc. 443.93).

##### Synthesis of 2-[4-(2-chloroethyl)piperazin-1-yl]-4-methoxy-6-(NH-Ala-Ala-OMe)-1,3,5-triazine (7b)

Starting material: 2-chloro-4-methoxy-6-(NH-Ala-Ala-OMe)-1,3,5-triazine (**4b**) (0.318 g, 1 mmol), DABCO (**5**) (0.112 g, 1 mmol). Product: 0.419 g 2-[4-(2-chloroethyl)piperazin-1-yl]-4-methoxy-6-(NH-Ala-Ala-OMe)-1,3,5-triazine (**7b**), yield = 97.4%, colorless oil.

Analysis: for C_17_H_28_ClN_7_O_4_ (429.91): calculated: C 47.5%, H 6.6%,Cl 8.2%, N 22.8%, found: C 47.6%, H 6.7%,Cl 8.2%, N 22.7%.

^1^H-NMR (250 MHz; CDCl_3_) δ = 1.37 (d, 3H, J = 7.0 Hz, CH_3_-CH-); 1.41 (d, 3H, J = 7.2 Hz, CH_3_-CH-); 2.66 (t, 2x2H, J = 7.8 Hz, -N-CH_2_-); 2.85 (t, 2H, J = 6.2 Hz, -N-CH_2_-); 3.60 (t, 2x2H, J = 7.8 Hz, -N-CH_2_-); 3.86 (2, 2H, J = 6.2 Hz, Cl-CH_2_-); 3.71 (s, 3H, CH_3_O-); 4.02 (s, 3H, CH_3_O-); 4.35 (q, 1H, J = 7.2 Hz, CH_3_-CH-); 4.38 (q, 1H, J = 7.0 Hz, CH_3_-CH-) [ppm].

^13^C-NMR (75 MHz, CDCl_3_): δ = 17.4, 17.7, 41.2, 44.0, 47.8, 49.2, 51.8, 52.3, 53.0, 163.4, 171.1, 172.1, 172.9 [ppm].

MS: 430.9118 ([M + H]^+^, C_17_H_29_ClN_7_O_4_+; calc. 429.91).

##### Synthesis of 2-[4-(2-chloroethyl)piperazin-1-yl]-4-methoxy-6-(NH-Ser-Ala-OMe)-1,3,5-triazine (7c)

Starting material: 2-chloro-4-methoxy-6-(NH-Ser-Ala-OMe)-1,3,5-triazine (**4c**) (0.334 g, 1 mmol), DABCO (**5**) (0.112 g, 1 mmol). Product: 0.438 g 2-[4-(2-chloroethyl)piperazin-1-yl]-4-methoxy-6-(NH-Ser-Ala-OMe)-1,3,5-triazine (**7c**), yield = 98.2%, colorless oil.

Analysis: for C_17_H_28_ClN_7_O_5_ (445.90): calculated: C 45.8%, H 6.3%, Cl 8%, N 22%, found: C 45.7%, H 6.4%, Cl 8%, N 22%.

^1^H-NMR (250 MHz; CDCl_3_) δ = 1.40 (d, 3H, J = 7.2 Hz, CH_3_-CH); 2.66 (t, 2x2H, J = 7.8 Hz, -N-CH_2_-); 2.85 (t, 2H, J = 6.2 Hz, -N-CH_2_-); 3.60 (t, 2x2H, J = 7.8 Hz, -N-CH_2_-); 3.68 (d, 2H, J = 6.8 Hz, -CH_2_-OH); 3.86 (2, 2H, J = 6.2 Hz, Cl-CH_2_-); 3.71 (s, 3H, CH_3_O-); 4.02 (s, 3H, CH_3_O-); 4.35 (q, 1H, J = 7.2 Hz, CH_3_-CH-); 4.47 (t, 1H, J = 6.8 Hz, -CH-CH_2_-OH) [ppm].

^13^C-NMR (75 MHz, CDCl_3_): δ = 17.6, 38.3, 41.2, 44.0, 47.8, 51.8, 52.3, 53.0, 54.6, 61.4, 163.5, 171.3, 171.7, 172.9 [ppm].

MS: 446.9342 ([M + H]^+^, C_17_H_29_ClN_7_O_5_^+^; calc. 445.90).

##### Synthesis of 2-[4-(2-chloroethyl)piperazin-1-yl]-4-methoxy-6-(NH-Lys(Boc)-Ala-OMe)-1,3,5-triazine (7d)

Starting material: 2-chloro-4-methoxy-6-(NH-Lys(Boc)-Ala-OMe)-1,3,5-triazine (**4d**) (0.475 g, 1 mmol), DABCO (**5**) (0.112 g, 1 mmol). Product: 0.580 g 2-[4-(2-chloroethyl)piperazin-1-yl]-4-methoxy-6-(NH-Lys(Boc)-Ala-OMe)-1,3,5-triazine (**7d**), yield = 98.8%, colorless oil.

Analysis: for C_25_H_43_ClN_8_O_6_ (587.11): calculated: C 51.1%, H 7.4%, Cl 6%, N 19.1%, found C 51.1%, H 7.4%, Cl 6%, N 19.1%.

^1^H-NMR (250 MHz; CDCl_3_) δ = 1.26–1.57 (m, 4H, -CH_2_-CH_2_-); 1.41 (d, 3H, d, J = 7.2 Hz, CH_3_-CH-); 1.43 (s, 9H, (CH_3_)_3_C-); 1.93 (dt, 2H, J_1_ = 7.5 Hz, J_2_ = 5.1 Hz, -CH-CH_2_-); 2.66 (t, 2x2H, J = 7.8 Hz, -N-CH_2_-); 2.85 (t, 2H, J = 6.2 Hz, -N-CH_2_-); 3.22 (t, 2H, J = 7.1 Hz, -CH_2_-NH-); 3.60 (t, 2x2H, J = 7.8 Hz, -N-CH_2_-); 3.86 (2, 2H, J = 6.2 Hz, Cl-CH_2_-); 3.71 (s, 3H, CH_3_O-); 4.02 (s, 3H, CH_3_O-); 4.28 (t, 1H, J = 7.5 Hz, -CH-CH^2^-); 4.36 (q, 1H, J = 7.2 Hz, CH_3_-CH-) [ppm].

^13^C-NMR (75 MHz, CDCl_3_): δ = 17.7, 21.4, 28.2, 29.3, 29.9, 40.5, 41.2, 44.0, 47.8, 51.8, 52.3, 53.1, 53.6, 54.6, 80.1, 156.2, 162.2, 171.7, 172.5, 172.9 [ppm].

MS: 588.2016 ([M + H]^+^, C_25_H_44_ClN_8_O_6_^+^; calc. 587.11).

##### Synthesis of 2-[4-(2-chloroethyl)piperazin-1-yl]-4-methoxy-6-(NH-Asp(OtBu)-Ala-OMe)-1,3,5-triazine (7e)

Starting material: 2-chloro-4-methoxy-6-(NH-Asp(OtBu)-Ala-OMe)-1,3,5-triazine (**4e**) (0.418 g, 1 mmol), DABCO (**5**) (0.112 g, 1 mmol). Product: 0.520 g 2-[4-(2-chloroethyl)piperazin-1-yl]-4-methoxy-6-(NH-Asp(OtBu)-Ala-OMe)-1,3,5-triazine (**7e**), yield = 98.2%, colorless oil.

Analysis: for C_22_H_36_ClN_7_O_6_ (530.02): calculated: C 49.9%, H 6.8%, Cl 6.7%, N 18.5%, found: C 49.8%, H 6.7%, Cl 6.7%, N 18.5%.

^1^H-NMR (250 MHz; CDCl_3_) δ = 1.38 (s, 9H, (CH_3_)_3_C-); 1.41 (d, 3H, J = 7.2 Hz, CH_3_-CH-); 3.01 (d, 2H, J = 6.3 Hz, -CH-CH_2_-); 2.66 (t, 2x2H, J = 7.8 Hz, -N-CH_2_-); 2.85 (t, 2H, J = 6.2 Hz, -N-CH_2_-); 3.60 (t, 2x2H, J = 7.8 Hz, -N-CH_2_-); 3.86 (2, 2H, J = 6.2 Hz, Cl-CH_2_-); 3.71 (s, 3H, CH_3_O-); 4.02 (s, 3H, CH_3_O-); 4.36 (1, 1H, J = 7.2 Hz, CH_3_-CH-); 5.02 (t, 1H, J = 6.3 Hz, -CH-CH_2_-) [ppm].

^13^C-NMR (75 MHz, CDCl_3_): δ = 17.7, 37.9, 41.1, 44.0, 47.8, 51.8, 52.3, 53.1, 53.5, 54.6, 81.1, 162.2, 171.7, 172.1, 172.5, 172.9 [ppm].

MS: 531.1256 ([M + H]^+^, C_22_H_37_ClN_7_O_6_^+^; calc. 530.02).

##### Synthesis of 2-[4-(2-chloroethyl)piperazin-1-yl]-4-methoxy-6-(NH-Trp(Boc)-Ala-OMe)-1,3,5-triazine (7f)

Starting material: 2-chloro-4-methoxy-6-(NH-Trp(Boc)-Ala-OMe)-1,3,5-triazine (**4f**) (0.533 g, 1 mmol), DABCO (**5**) (0.112 g, 1 mmol). Product: 0.638 g 2-[4-(2-chloroethyl)piperazin-1-yl]-4-methoxy-6-(NH-Trp(Boc)-Ala-OMe)-1,3,5-triazine (**7f**), yield = 98.9%, colorless oil.

Analysis: for C_30_H_41_ClN_8_O_6_ (645.15): calculated: C 55.9%, H 6.4%, Cl 5.5%, N 17.4%, found: C 55.9%, H 6.4%, Cl 5.5%, N 17.4%.

^1^H-NMR (250 MHz; CDCl_3_) δ = 1.41 (d, 3H, J = 7.2 Hz, CH_3_-CH-), 1.44 (9H, s, (CH_3_)_3_-C-), 2.66 (t, 2x2H, J = 7.8 Hz, -N-CH_2_-); 2.85 (t, 2H, J = 6.2 Hz, -N-CH_2_-); 3.15 (d, 2H, J = 6.7 Hz, -CH_2_-CH-); 3.60 (t, 2x2H, J = 7.8 Hz, -N-CH_2_-); 3.86 (2, 2H, J = 6.2 Hz, Cl-CH_2_-); 3.72 (s, 3H, CH_3_O-); 4.02 (s, 3H, CH_3_O-); 4.36 (q, 1H, J = 7.2 Hz, CH_3_-CH-); 4.91 (t, 1H, J = 6.7 Hz, -CH_2_-CH-); 7.03–7.98 (m, 5H, arom) [ppm].

^13^C-NMR (75 MHz, CDCl_3_): δ = 17.7, 27.7, 28.1, 41.2, 44.1, 47.9, 51.8, 52.3, 53.0, 53.6, 54.5, 81.1, 115,1, 117.1, 118.7, 120.6, 122.7, 123.7, 129.6, 135.4, 149.5, 163.3, 172.4, 172.9 [ppm].

MS: 646.15864 ([M + H]^+^, C_30_H_42_ClN_8_O_6_^+^; calc. 645.15).

### Biology

#### Reagents

RPMI-1640 medium, McCoy’s 5a medium, fetal bovine serum, penicillin and streptomycin were obtained from the American Type Culture Collection (ATCC) (Manassas, VA). 5-Fluorouracil (5-Fluorouracil-Ebewe) was obtained from Ebewe (Unterach, Austria).

#### Cell culture

Cells were purchased from ATCC. Cell line DLD-1 was cultured in RPMI 1640 medium (Sigma, USA), line HT-29 in McCoy’s 5a medium (Sigma, USA) supplemented with 10% fetal calf serum (Sigma, USA), penicillin (50 IU, Sigma, USA) and streptomycin (50 μg/l, Sigma, USA), in an incubator with 5% CO_2_ (normoxia), at a relative humidity of 95%, at 37 °C (Heraeus, USA). The culture media was changed every two days. The cells were generally kept in 75 cm^3^ flasks (Sarstedt, USA). However, for the experiments the cells were plated in 100-mm dishes (Sarstedt, USA) with 10 ml of medium. The control was medium with PBS only.

#### Drug treatment

Prior to each treatment, DLD-1 and Ht-29 cells were grown until they reached 50–60% confluence. The cells were then treated with 5-FU and **7b** at different concentrations (1, 5, 10, 50 μM) for 24 or 48 h. Untreated cells were used as the control.

#### Proliferation assay

A proliferation assay was performed according to the method described by Mosmann using 3-(4,5-dimethylthiazol-2-yl)-2,5-diphenyltetrazolium bromide (MTT) [26]. Confluent cells cultured in 6-well plates for 24 or 48 h with various concentrations of the studied compounds **7a**-**7 f.** The plates were washed three times with PBS and then incubated for 4 h in 1 ml of MTT solution (0.5 mg/ml of PBS) at 37 °C in a 5% CO_2_ incubator. The medium was removed and 1 ml of 0.1 M HCl in absolute isopropanol was added to the attached cells. The absorbance of the converted dye in the living cells was measured at a wavelength of 570 nm. The viability of DLD-1 and HT-29 cells cultured in the presence of the studied compounds was calculated as a percentage of the control cells. The experiments were performed in triplicate. The ratio of living to dead cells with and without the drugs (control) was calculated for each drug concentration.

#### Quantitative assessment of apoptotic cells

Colon and breast cells were stained with annexin-V-Fluos and propidium iodide (FITC Annexin V Apoptosis Detection Kit I BD, Pharmingen). After 10 min of incubation in ice and darkness, the samples were analyzed with a FACSCalibur flow cytometer (BD). The results were verified by microscopy (Olympus CH30) (Annexin V-FITC Fluorescence Microscopy Kit BD, Pharmingen), counting 100 consecutive cells at 200× magnification. The percentage of apoptotic cells was calculated based on the average taken from both study methods.

#### Analysis of mitochondrial membrane potential

Dysfunction of the mitochondrial membrane potential (MMP) was assessed using the lipophilic cationic probe 5,5′,6,6′-tetrachloro-1,1′,3,3′-tetraethylbenzimidazol-carbocyanine iodide (JC-1 MitoScreen kit; BD Biosciences). Briefly, unfixed cells were washed and resuspended in PBS supplemented with 10 μg/ml JC-1. The cells were then incubated for 15 min at room temperature in darkness, washed again and resuspended in PBS for immediate BD FACSCanto II flow cytometry analysis (Bioscences Systems, CA). The percentage of cells with disrupted MMP was calculated using FACSDiva software (Bioscences Systems, CA).

#### Western blot analysis for Bax and Bcl-2

Cells were lysed in NP-40 buffer (50 mM Tris-HCl (pH 8.0), 150 mM NaCl, 1% Triton X-100 and protease inhibitor cocktail (Roche)). The lysate was centrifuged at 10000×g for 20 min at 4 °C. An aliquot (10 μl) of the supernatant was subjected to electrophoresis in a 10% SDS-polyacrylamide gel, then transferred to a nitrocellulose membrane (Bio-Rad), pore size 0.2-μm, according to the method described in the manual accompanying the unit. The blots were blocked for 1 h at room temperature with 5% nonfat milk (Bio-Rad) in Tris-buffered saline, pH 8.0 (Sigma-Aldrich, St. Louis, MO). The membrane was incubated with mouse anti-Bax (1:400), mouse anti-Bcl-2 (1:400) or mouse anti-*β*-actin (1:3000) antibodies from Sigma-Aldrich (St. Louis, MO) in TBS-T (20 mM Tris-HCl buffer (pH 7.4) containing 150 mM NaCl and 0.05% Tween 20) overnight. Alkaline phosphatase-conjugated goat anti-mouse secondary antibodies (Sigma-Aldrich, St. Louis, MO) were added at a 1:10000 dilution in TBS-T and incubated for 1 h with slow shaking. The nitrocellulose was then washed with TBS-T (2 × 10 min) and exposed to the Sigma-Fast BCIP/NBT reagent. The intensity of the bands was quantified by densitometric analysis using Image J 1.45 software (National Institute of Health, USA).

#### Statistical analysis

The normally distributed data were presented as mean ± SD. Normality of distribution was tested using the Shapiro–Wilk test. Multiple group comparisons were performed by one-way analysis of variance and significant differences between groups were assessed by the Tukey–Kramer test. Computations were performed using GraphPad 7 Prism software (GraphPad Software, Inc., La Jolla, CA, USA).

## Results

### Preparation of new analogues of nitrogen mustard derivatives containing dipeptide substituents on 1,3,5-triazine ring **7a-f**

**Scheme 1:**
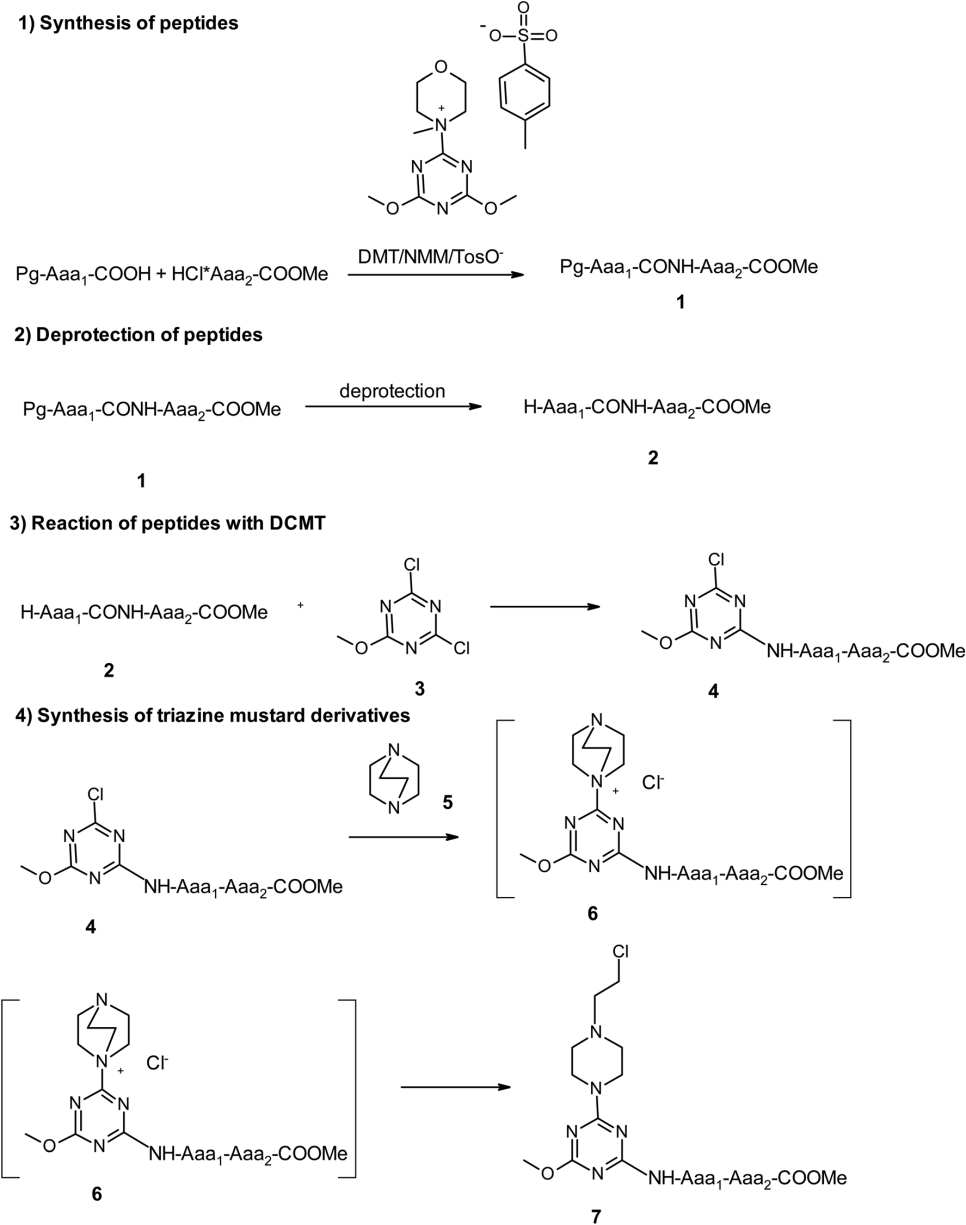
Multi-stage synthesis of nitrogen mustard derivatives **7a-f** containing dipeptide substituents on the 1,3,5-triazine ring

**Fig. 2:**
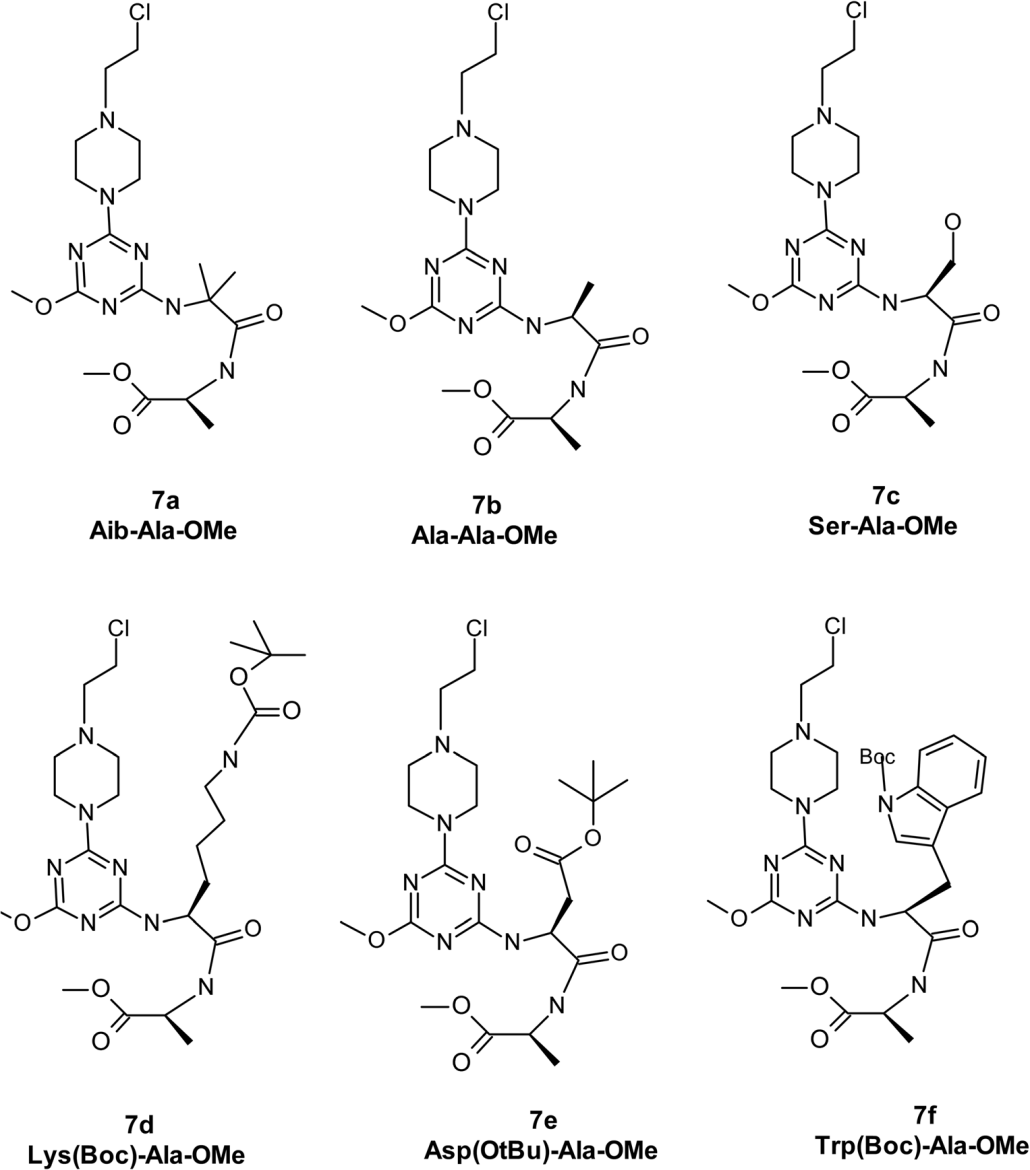
Structures of the new triazine-peptide-mustards **7a-f**

The synthesis of new analogues of nitrogen mustards **7a-f** containing the 1,3,5-triazine ring substituted with dipeptide residue is a multi-step process (Scheme 1). The first stage is the synthesis of dipeptides **1**. This was made in a solution using 4-(4,6-dimethoxy-1,3,5-triazin-2-yl)-4-methylmorpholinium toluene-4-sulfonate (DMT/NMM/TosO^−^) [27] as a coupling reagent. After removing the protective groups from the amine function, the dipeptide derivatives **2** were reacted with 2,4-dichloro-6-methoxy-1,3,5-triazine (DCMT, **3**) in the presence of solid NaHCO_3_ as a hydrogen chloride acceptor. Derivatives **4** were obtained with yields ranging from 95.8% to 98.7%. Due to the presence of halogen on the ring of 1,3,5-triazine, derivatives **4** undergo quaternization with tertiary amines, leading to the formation of triazinylammonium chloride **6**. However, due to their impermanence, they are easily dealkylated [28, 29]. Use of DABCO as a tertiary amine enables derivatives containing the 2-chloroethylamine substituent characteristic of nitrogen mustard to be obtained as a result of the bicyclic ring opening process [24, 25]. Finally, six new analogues of nitrogen mustards **7** with different dipeptide substituents on the 1,3,5-triazine ring were prepared (Fig. 2).

### Triazine derivatives decrease colon cancer viability

**Fig. 3:**
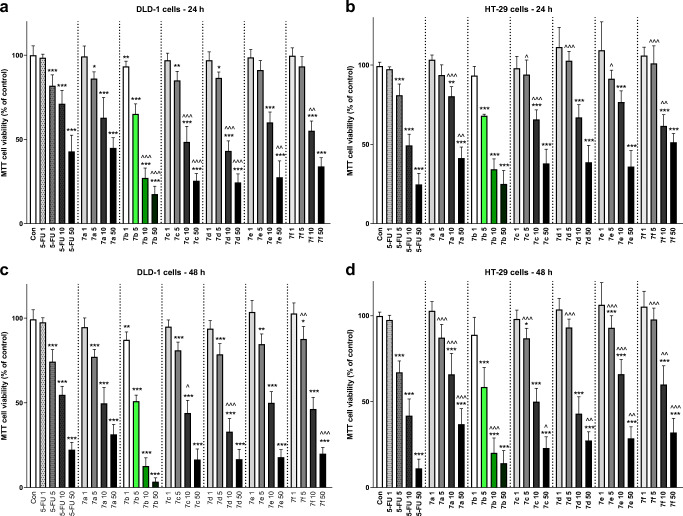
Effects of 5-fluorouracil (**5-FU**) and triazine derivatives **7a-b** on the viability of DLD-1 (**a**, **c**) and HT-29 (**b**, **d**) cells after 24 h (**a**, **b**) or 48 h (**c**, **d**) of incubation. Results are presented as means ± SD, *n* = 6, *P < 0.05 (vs. Con), ^P < 0.05 (vs. 5-FU)

In the first stage of our in vitro study, we investigated the effective cytotoxic dose of triazine derivatives. The MTT test indicated that all of the compounds exhibited cytotoxic effects, but there were differences in the sensitivity of colon cancer cells (Fig. 3). Prolonged incubation for 48 h increased the cytotoxic effects of all the compounds.

Of all the synthesized compounds, **7b** showed the highest activity against cells from both lines. Therefore, we used this derivate in further research. The DLD-1 cell lines were found to be more sensitive to compound **7b** than the HT-29 line. The IC_50_ values for **7b** were at least 2-fold lower than those for **5-FU** in both tested lines after 48 h of incubation (Table 1).

**Table 1:** Antiproliferative activity of 5-fluorouracil (**5-FU**) and compounds **7a-f**

	IC_50_ [μM]	
	DLD-1	HT-29
5-FU	27.22	21.72
7a	30.27	36.46
7b	13.71	17.78
7c	23.80	28.06
7d	24.54	29.54
7e	26.33	32.85
7f	26.85	34.02

### Triazine derivative induces apoptosis through intracellular signaling pathway attenuation

**Fig. 4:**
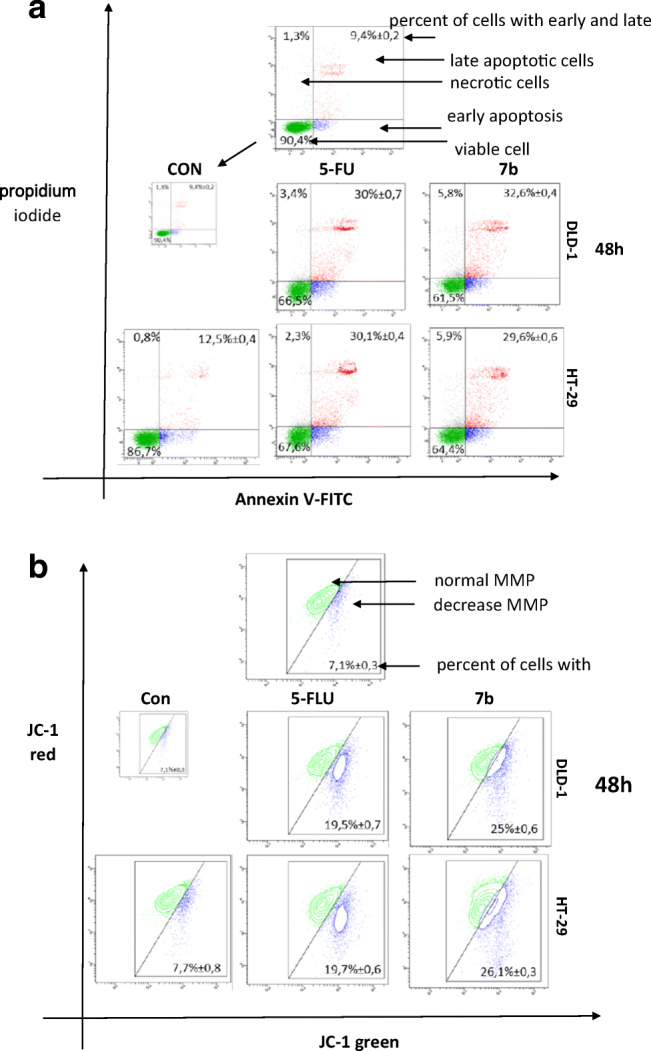
Representative flow cytometry dot-plots for Annexin V-FITC assay of DLD-1 and HT-29 cells incubated with **7b** (10 μM) for 48 h (mean ± SD; *n* = 3). Live cells appear in the lower left corner of the plots. Early apoptotic cells appear in the lower right corner. Necrotic cells appear in the upper-left corner. Dead cells appear in the upper-right corner (**a**). Representative dot-plots presenting the loss of mitochondrial membrane potential (MMP) of DLD-1 and HT-29 cells incubated with **7b** (10 μM) for 48 h (mean ± SD; n = 3). Cells with normal MMT are shown on the right-hand side of the plots, cells with decreased MMT on the left-hand side of the plots (**b**)

To quantitate the extent of apoptosis, the cells were *stained* with annexin V, a phospholipid-binding protein that has high affinity for phosphatidylserine (PS) and enables identification of cells in the early stages of apoptosis. Propidium iodide (PI) was used to identify late apoptotic and dead cells. Dual-labeling annexin V/propidiumiodide (PI) allowed us to assess the apoptosis status of DLD-1 and HT-29 cells after 48 h of **7b** and 5-FU treatment. We observed that the tested compound significantly induced programmed cell death in DLD-1 and HT-29 cells in comparison to the control (untreated cells). We also found that the pro-apoptotic potential of **7b** was similar to that of 5-FU. For 5-FU and **7b** in DLD-1**,** we observed 61.5% and 66.5% viable cells and 32.6% and 30.0% apoptotic cells, respectively. For 5-FU and **7b** in HT-29, we observed 64.4% and 67.6% viable cells and 29.6% and 30.1% apoptotic cells, respectively (Fig. 4a).

In order to investigate the cellular mechanism underlying **7b**-induced intrinsic apoptosis in DLD-1 and HT-29 using flow cytometry analysis and lipophilic fluorochrome JC-1, we estimated the alterations of mitochondrial transmembrane potential (MMP). As shown in Fig. 4b, **7b** increased the number of cells with decreased levels of MMP in both DLD-1 (25 ± 0.6%) and HT-29 (26,1 ± 0.3%) cells after 48 h of incubation. For untreated cells, the decrease in mitochondrial potential was 7.1% in DLD-1 and 7.7% in HT-29. We observed that the decreases in MMP caused by **7b** and 5-FU were similar. These results are in line with those obtained in the Annexin-V and PI assay.

**Fig. 5:**
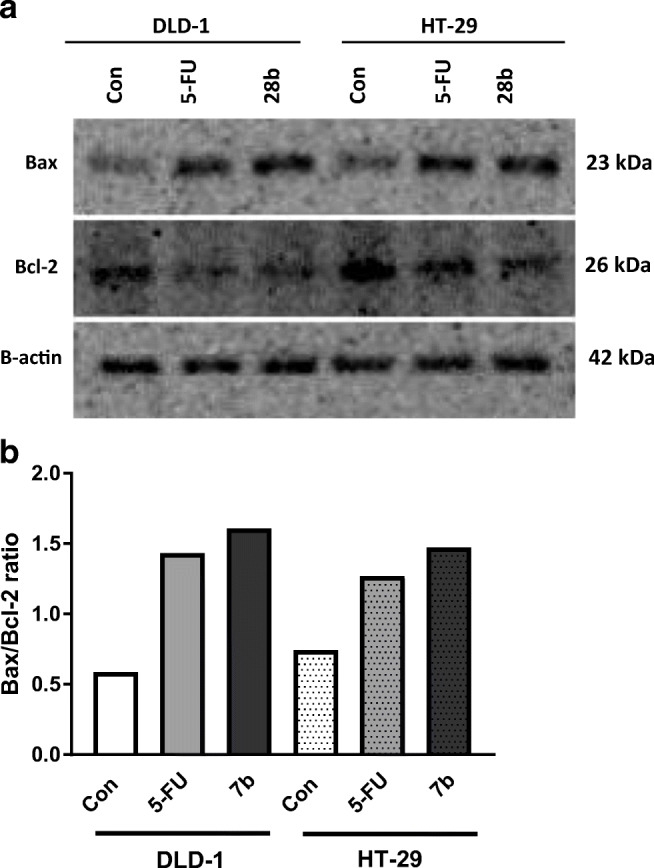
Immunoblotting analysis for BAX and Bcl-2 in DLD-1 and HT-29 cells treated with **7b** (10 μM) for 48 h (**a**). The samples used for electrophoresis consisted of 20 μg of protein from six pooled cell extracts (*n* = 6). *Bax*/*Bcl*-*2* protein expression was performed using a scanning *densitometer* (**b**)

Treatment for 48 h with **7b** contributed to a marked increase in the expression of BAX compared with control cells in both the DLD-1 and HT-29 lines. We observed a decrease in the expression of BCL-2 after incubation with **7b** compared with the control cells. Incubation with **7b** led to an increase of BCL-2 expression both in DLD-1 and in HT-29 cells. This effect was more pronounced than after incubation of cells with 5-FU. An increase in the BAX/BCL-2 ratio was linked with increased apoptosis. The ratio of BAX to BCL-2 shifted in favor of BAX compared with the control, which confirms the intensification of this process (Fig. 5).

## Conclusion

Hybrid compounds based on nitrogen mustard 1,3,5-triazine have great therapeutic potential, as confirmed by numerous reports, including our research. In this paper, we reported the synthesis a new series of trisubstituted triazine derivatives. The investigated compounds **7a**-**f** displayed an interesting spectrum of activity. All of them exhibited cytotoxic effects against DLD-1 and HT-29 colon cancer cells. Compound **7b**, in which the 1,3,5-triazine ring was substituted with an Ala-Ala-OMe group and one 2-chloroethylamino moiety, was the most active, with IC_50_ values of 13.71 μM. This was at least 2-fold lower than the values for **5-FU,** for which the IC_50_ was equal to 27.22 μM. Because of its activity, the mechanism of **7b** action was examined in detail. It was determined that this triazine derivative induces apoptosis through intracellular signaling pathway attenuation.

Our research confirms the value of triazine derivatives as potential anticancer agents. Further investigations are needed to ascertain whether the studied compounds should be considered for possible therapeutic applications.
